# Level of Effort: A Practical Approach for Resistance Training Monitoring and Prescription

**DOI:** 10.5114/jhk/221229

**Published:** 2026-04-30

**Authors:** Fernando Pareja-Blanco, Irineu Loturco

**Affiliations:** 1 Science-Based Training Research Group, Physical Performance and Sports Research Center, Universidad Pablo de Olavide, Seville, Spain.; 2 Faculty of Sports Sciences, Department of Sports and Computer Sciences, Universidad Pablo de Olavide, Seville, Spain.; 3 NAR—Nucleus of High Performance in Sport, São Paulo, Brazil.; 4 Department of Human Movement Sciences, Federal University of São Paulo, São Paulo, Brazil.; 5 FSI—Football Science Institute, Granada, Spain.

**Keywords:** athletic performance, muscle strength, strength training, fatigue, velocity-based training

## Abstract

Accurate prescription and monitoring of resistance training (RT) load require ensuring that the external load prescribed by the coach reflects the internal demands experienced by the athlete. Although repetitions in reserve (RIR) have been proposed as a practical method for quantifying proximity to failure, their use in isolation assumes that identical RIR values correspond to equivalent physiological stimuli across different repetition ranges and intensities. This assumption does not necessarily reflect comparable internal loading conditions. To address this limitation, the concept of “level of effort”—defined as the “relationship between the repetitions performed and the maximum number of repetitions that could be completed with a given load”—offers a proportion-based and more comprehensive representation of exertion. When quantified through movement velocity, the level of effort integrates two critical variables: relative intensity (via the fastest repetition velocity) and fatigue development (via velocity loss), allowing accurate estimation of the percentage of repetition capacity utilized within a set. This approach enables practitioners to derive RIR from an objective measure of actual effort, thereby enhancing the precision of RT prescription and monitoring.

## Introduction

Training load refers to the input applied during training that elicits a physical and physiological response from the athlete (Impellizzeri et al., 2019). Training load can be described as prescribed (external) or actual (internal), depending on whether it refers to the work performed or the psychophysiological response elicited (Impellizzeri et al., 2019). The prescribed, or external, load is defined as the physical work specified in the training plan, including exercise selection, intensity, volume, and other loading variables (e.g., rest intervals between sets). The internal load refers to the psychophysiological response elicited within the athlete to cope with the demands imposed by the external load (Impellizzeri et al., 2019). The planned load corresponds to the intended internal load—that is, the level of stress targeted for the individual—yet it is operationally represented by the prescribed load.

During a training program, two of the main tasks of the coach are to: 1) ensure that the planned load is appropriate to produce the desired adaptations, and 2) ensure that the prescribed load accurately reflects the actual load. Failure to accurately align prescribed and actual loads may compromise progress within the training process, as the adaptations achieved will depend on the stimulus actually imposed rather than the one theoretically planned (Kraemer and Ratamess, 2004; Zatsiorsky et al., 2021). When the relationship among the external load (i.e., what is prescribed and performed), the internal load (i.e., the physiological and neuromuscular stress imposed), and the resulting adaptations is not objectively verified, the training process becomes a “black box”. Thus, objective monitoring strategies are essential to close this gap, ensuring a transparent and measurable link between prescription, execution, and chronic adaptations. To achieve this goal, it is essential to have a deep understanding of the two main components of training load: 1) intensity, usually described in relation to an objective or subjective maximum value of a given variable; and 2) volume, often expressed in units of time, distance, or the number of repetitions.

Training intensity is considered one of the most important variables when designing a resistance training (RT) program (Kraemer and Ratamess, 2004; Zatsiorsky et al., 2021). To individualize and facilitate programming, training intensity is commonly expressed relative to each athlete’s maximal capacity (Suchomel et al., 2018). In RT, relative intensity is typically expressed as a percentage of an individual’s one-repetition maximum (%1RM) (Cormie et al., 2011; Stone et al., 2007), that is, the maximum load an individual can lift during a maximal effort in a specific exercise (e.g., bench press or squat exercises). Nevertheless, the training effect of an RT session is determined by both the magnitude of relative intensity (Schoenfeld et al., 2017) and the total volume performed (sets × repetitions) (Rhea et al., 2003; Stone et al., 1999).

Specifically, training volume is intended to quantify the amount of work performed (Kraemer and Ratamess, 2004). This variable plays a central role in determining the responses and adaptations induced by RT (Grgic et al., 2022; Wernbom et al., 2007). However, as with intensity, the value of volume alone is insufficient to define the RT load, since the same volume yields a very different training stimulus when intensity varies. Several approaches have been used to determine RT volume. The most basic approach to quantifying training volume is the use of a repetition-based protocol, defined as the total number of repetitions performed during a given training session (Kraemer and Ratamess, 2004). While the simplicity of this procedure is advantageous for group settings, it is insufficient for accurately monitoring the internal training load experienced by each athlete. The main problem with this method is that it primarily considers external loads without providing accurate information about the fatigue they induce across athletes within a given training session (Halson, 2014; Impellizzeri et al., 2019). This complicates one of the coach’s main tasks: determining the actual load imposed on the athlete.

A recent consensus statement by the American College of Sports Medicine (ACSM) and Exercise & Sport Science Australia (ESSA) emphasizes the importance of considering progression toward neuromuscular failure when prescribing RT intensity, as fatigue accumulates with repetitions, especially near failure (Bishop et al., 2025). This consensus statement proposes the use of repetitions in reserve (RIR) as an alternative approach to training load prescription for both novice and experienced lifters (Bermúdez Droguett et al., 2025). The term of RIR was initially defined by Zourdos et al. (2016) as “the number of additional repetitions an individual believes they could complete before reaching muscular failure”. Despite the importance of considering proximity to task failure, the use of RIR as a standalone indicator presents several limitations that should be considered.

One key concern is the assumption that a given RIR value reflects a comparable training stimulus across different repetition ranges. This assumption implicitly treats proximity to failure as the sole determinant of exertion, disregarding the relative proportion of repetitions performed in relation to the total number of repetitions possible. This discrepancy may influence the magnitude of metabolic stress, neuromuscular fatigue, and subsequent recovery demands (González-Badillo and Sánchez-Medina, 2010; Sánchez-Medina and González-Badillo, 2011; Schoenfeld, 2010). For instance, equating RIR of 2 when only 2 repetitions are performed with RIR of 2 after 10 repetitions completed can be misleading: in the first case, the athlete completes 50% of the possible repetitions (2 of 4), whereas in the second, they complete approximately 83% (10 of 12). While both conditions technically result in a “two-repetition reserve”, the underlying physiological demands are quite distinct. Supporting this, Sánchez-Medina and González-Badillo (2011) reported marked differences in metabolic and mechanical stress during the squat exercise, including blood lactate (3.0 vs. 10.6 mmol•L^−1^), blood ammonia (41 vs. 62 µmol•L^−1^), jump height loss (5.7% vs. 16.6%), and strength impairment (6.1% vs. 14.6% for the 2 (of 4) and 10 (of 12) protocols, respectively).

In summary, these findings indicate that identical RIR values can correspond to different levels of accumulated fatigue and internal stress, thereby challenging the assumption that RIR alone can standardize the training stimulus across varying repetition configurations. From a programming perspective, this inconsistency may contribute to variability in fatigue accumulation and subsequent adaptive responses when similar RIR targets are prescribed under different loading schemes.

## The Level of Effort Concept

The term “level of effort” was originally defined by González-Badillo and Gorostiaga (1995) and later empirically examined by Sánchez-Medina and González-Badillo (2011). It describes the work performed by an individual relative to their maximum strength capacity; that is, the relationship between the number of repetitions performed in a set and the total number of repetitions achievable (González-Badillo and Gorostiaga, 1995; Sánchez-Medina and González-Badillo, 2011). This concept expresses exertion proportionally, providing a more consistent representation of training demands based on the proportion of repetition capacity utilized within a set. Hence, the level of effort addresses the limitations of RIR by simultaneously considering two components: (a) the difference between repetitions performed and the maximum number of repetitions possible (i.e., RIR), and (b) the total repetition capacity associated with a given load. Accordingly, RIR represent only one component of the effort construct, as they reflect distance to failure without accounting for the relative workload completed within a respective set (González-Badillo et al., 2017).

Returning to the previous example, completing 2 (of 4) and 10 (of 12) repetitions yields the same difference of 2 repetitions between those performed and those possible, yet the acute physiological responses are considerably different. Although the level of proximity to failure is identical, the total repetition capacity differs substantially, thereby influencing acute fatigue, metabolic stress, and both central and peripheral fatigue responses (Sánchez-Medina and González-Badillo, 2011). This distinction is critical because physiological stress is not determined solely by how many repetitions remain, but by how much of the available repetition capacity has already been utilized. Therefore, a proportion-based representation of exertion more accurately reflects the internal load imposed during a set (Pareja-Blanco et al., 2017; Sánchez-Medina and González-Badillo, 2011) (Table 1).

**Table 1:** Comparison between repetitions in reserve (RIR) and proportional level of effort across different repetition scenarios.

Scenario	Repetitions Performed	Maximum Possible Repetitions	RIR (Repetitions Remaining)	Level of Effort (% Repetitions Capacity Completed)
**A**	**2**	**4**	**2**	**2 of 4 (50%)**
**B**	**10**	**12**	**2**	**10 of 12 (83%)**
**C**	**4**	**16**	**12**	**4 of 16 (25%)**
**D**	**8**	**16**	**8**	**8 of 16 (50%)**
**E**	**9**	**10**	**1**	**9 of 10 (90%)**
**F**	**4**	**5**	**1**	**4 of 5 (80%)**

In essence, the level of effort provides a more precise representation of the exertion imposed by a given load and better reflects the internal demands placed on the athlete. By integrating both the number of remaining repetitions and total repetition capacity, it establishes a standardized framework for interpreting training stress across varying repetition ranges and loading intensities, thereby overcoming the conceptual and structural limitations inherent in RIR when used in isolation.

## Level of Effort Defined by Lifting Velocity

Currently, the level of effort can be accurately quantified through velocity-based measurements. The initial indicator of effort is determined by the difficulty of the first repetition; that is, by the relative loading intensity. Consequently, the level of effort is initially established by the velocity of the fastest repetition (typically the first or the second), which allows for a highly accurate estimation of the percentage of the 1RM (González-Badillo and Sánchez-Medina, 2010). To ensure a valid interpretation of effort, each repetition must be performed with maximal intent, as submaximal execution velocities fail to precisely reflect the true relative intensity of the load (Pareja-Blanco et al., 2014).

However, this alone does not fully define the level of effort, as overall exertion also depends on the proportion of the maximum number of possible repetitions performed: completing 1 repetition with a load that allows for 10 repetitions [1 (of 10)] is not equivalent to completing 8 repetitions with that same load [8 (of 10)]. Given that performing repetitions at maximal velocity induces a progressive decline in movement velocity, the level of effort is determined not only by the difficulty of the first repetition, but also by the velocity loss (VL) throughout the set (Sánchez-Medina and González-Badillo, 2011). As such, VL serves as an objective proxy for the proportion of repetition capacity utilized, providing a quantifiable expression of the proportion-based construct previously described (Bachero Mena et al., 2025; González-Badillo et al., 2017). The integration of fastest repetition velocity and intra-set VL represents a practical evolution of the level of effort construct (Pareja-Blanco et al., 2017, 2020).

By measuring VL within a set, it is also possible to estimate effort without knowledge of the total number of repetitions that could be performed, as strong relationships (R^2^ = 0.92–0.97) have been observed between VL and the percentage of repetitions completed relative to the maximum achievable (%Rep, i.e., proximity to muscle failure) (González-Badillo et al., 2017; Sánchez-Moreno et al., 2021). Moreover, the %Rep completed at a given VL magnitude exhibits low variability (inter-individual coefficient of variation [CV]: 2.5–12.1%) and high reliability (intra-individual CV: 2.1–6.6%) (González-Badillo et al., 2017). Therefore, the %Rep corresponding to a given magnitude of VL appears to be relatively consistent across individuals, regardless of their maximum number of repetitions (González-Badillo et al., 2017; Sánchez-Moreno et al., 2021). This consistency supports the use of VL as a standardized indicator of intra-set effort (Bachero Mena et al., 2025; Rodiles-Guerrero et al., 2026). Conversely, the concept of RIR relies solely on the absolute number of repetitions remaining and does not account for differences in total repetition capacity (Helms et al., 2016; Zourdos et al., 2016).

This distinction is crucial for training prescription, as similar RIR values do not guarantee comparable physiological stress. It is important to emphasize that the present proposal does not aim to disregard the practical value of RIR in training prescription. On the contrary, RIR remain a widely adopted and useful method, and many RT programs can be designed with considerable precision using this strategy. Our intention is not to replace the RIR method, but rather to offer an alternative conceptual framework that enables coaches to define and monitor RT loads with greater proportional precision. By incorporating the level of the effort approach, strength and conditioning coaches may further enhance the individualization, effectiveness, and physiological precision of RT prescription.

## Practical Implications

As a general guideline, and recognizing that effort exists on a continuum, the level of effort is considered low when the number of repetitions performed is far from the maximum achievable. In terms of intra-set VL, this typically corresponds to ~5–10% relative to the fastest repetition. Hence, this reflects a high initial velocity and minimal VL, with the repetitions performed generally representing less than half of the total capacity. As examples of low effort, the following values may be considered: 4–6 (of 16–30) or 3–4 (of 10–14). Although both scenarios are classified as “light”, their physiological impact differs considerably and would be prescribed in different contexts. From an applied perspective, interpreting effort relative to total capacity and VL may provide a more accurate reflection of the internal load than relying solely on RIR (Bastos et al., 2024; Mansfield et al., 2020).

The level of effort is considered moderate when approximately half of the possible repetitions are completed, corresponding to a VL of ~15–25%—magnitude associated with intermediate neuromuscular fatigue (Rodríguez-Rosell et al., 2020; Sánchez-Medina and González-Badillo, 2011); for example: 6–7 (of 12–14) or 4–5 (of 8–10) repetitions completed. Effort is categorized as high or very high when more than half of the possible repetitions are performed, implying a VL exceeding ~25–30%, yet with 2–4 repetitions remaining; for example: 3 (of 5), 4 (of 7), 5–6 (of 8), or 8 (of 12) repetitions completed. Effort is maximal when the maximum or near-maximum number of repetitions is completed, with VL reaching very high levels (50–70%), as illustrated by 9–10 (of 10), 7–8 (of 8), or 3–4 (of 4) repetitions completed. These categories are not rigid thresholds but practical reference points in which the magnitude of VL rather than the number of repetitions remaining dictates the internal stress imposed (Rodiles-Guerrero et al., 2026).

Nevertheless, these guidelines are general because, within this framework, when movement velocity is measured, the target is not a fixed number of repetitions, but rather the VL to be reached with a given load. This approach results in a different number of repetitions across individuals for the same level of effort. Such inter-individual variability is not a limitation but a core strength of this model, as it ensures exposure to equivalent levels of neuromuscular stress despite differences in repetition capacity, fiber composition, or fatigue resistance (Martinez-Canton et al., 2021; Pareja-Blanco et al., 2017). In contrast, prescribing identical RIR targets may lead to substantial discrepancies in accumulated fatigue and recovery demands. It is important to acknowledge that this is a conceptual article including an objective proposal, which should be tested in future studies comparing the two distinct methods (i.e., RT prescription based on RIR or level of effort approaches) and the potential neuromuscular adaptations provided by each of these methods. In addition, the dependence on linear position or velocity transducers to facilitate the implementation of the suggested approach may limit its application in less-equipped training facilities. Lastly, due to differences in training backgrounds and performance levels among athletes, the external validity of the proposed VL thresholds may be limited; therefore, these thresholds might need to be adjusted on an individual basis.

## Conclusions

In conclusion, relying solely on RIR may provide an incomplete representation of the actual exertion imposed during RT, as it disregards the total number of possible repetitions, which fundamentally modulates the physiological stimulus. The level of effort approach addresses this limitation by integrating both components of exertion—repetitions performed and repetitions possible—thereby providing a clearer and more precise understanding of the internal demands placed on the athlete. By adopting a proportion-based perspective, this construct overcomes the structural limitations inherent in methods that rely exclusively on the absolute number of repetitions remaining. This concept enhances the interpretation of proximity to failure and improves the standardization of training stress across diverse repetition ranges, loading schemes, and individual capacities. When available, velocity-based feedback serves as a robust tool to objectively quantify exertion, providing an empirically supported and low-variability indicator of the proportion of repetition capacity utilized. In applied training settings, this approach allows practitioners to derive RIR from an objective quantification of effort, rather than relying solely on RIR to infer exertion. As a construct, the level of effort represents a more comprehensive and informative metric than RIR alone. Adopting this perspective as a core principle in RT prescription enhances both scientific rigor and practical decision-making, enabling coaches and sport scientists to better align prescribed training with the intended adaptive stimulus. The level of effort provides a coherent, standardized, and physiologically grounded framework for prescribing and monitoring RT load.

## References

[ref1] Bachero Mena, B., Rodiles Guerrero, L., Sánchez Valdepeñas, J., Cornejo Daza, P. J., Cano Castillo, C., Pareja Blanco, F. & Sánchez Moreno, M. (2025). Velocity Loss as an Indicator of Resistance Training Volume in Women. *Journal of Human Kinetics*, 95, 111–122. 10.5114/jhk/190387 39944969 PMC11812168

[ref2] Bastos, V., Machado, S., & Teixeira, D. S. (2024). Feasibility and Usefulness of Repetitions-In-Reserve Scales for Selecting Exercise Intensity: A Scoping Review. *Perceptual and Motor Skills*, 131(3), 940–970. 10.1177/00315125241241785 38563729 PMC11127506

[ref3] Bermúdez Droguett, F. A., Ricardo Festa, R., Telchi Quintana, N. A., Gomez-Lopez, J., Huerta Ojeda, A., Farías Valenzuela, C., Giakoni-Ramírez, F. & Jofré-Saldía, E. (2025). Objective Accuracy in Estimating Repetitions in Reserve in the Back Squat: An Analysis between Experienced vs. Novice Subjects. *Journal of Human Kinetics*, Advance online publication. 10.5114/jhk/205218 PMC13215226 42211811

[ref4] Bishop, D. J., Beck, B., Biddle, S. J. H., Denay, K. L., Ferri, A., Gibala, M. J., Headley, S., Jones, A. M., Jung, M., Lee, M. J. C., Moholdt, T., Newton, R. U., Nimphius, S., Pescatello, L. S., Saner, N. J., & Tzarimas, C. (2025). Physical Activity and Exercise Intensity Terminology: A Joint American College of Sports Medicine (ACSM) Expert Statement and Exercise and Sport Science Australia (ESSA) Consensus Statement. *Journal of Science and Medicine in Sport*, 28(12), 980–991. 10.1016/j.jsams.2024.11.004 41093682

[ref5] Cormie, P., McGuigan, M. R., & Newton, R. U. (2011). Developing Maximal Neuromuscular Power: Part 2–Training Considerations for Improving Maximal Power Production. *Sports Medicine*, 41(2), 125–146. 10.2165/11538500-000000000-00000 21244105

[ref6] González-Badillo, J. J., & Gorostiaga, E. (1995). Fundamentos del Entrenamiento de la Fuerza. INDE publicaciones.

[ref7] González-Badillo, J. J., & Sánchez-Medina, L. (2010). Movement Velocity as a Measure of Loading Intensity in Resistance Training. *International Journal of Sports Medicine*, 31(5), 347–352. 10.1055/s-0030-1248333 20180176

[ref8] González-Badillo, J. J., Yañez-García, J. M., Mora-Custodio, R., & Rodríguez-Rosell, D. (2017). Velocity Loss as a Variable for Monitoring Resistance Exercise. *International Journal of Sports Medicine* , 38(3), 217–225. 10.1055/s-0042-120324 28192832

[ref9] Grgic, J., Schoenfeld, B. J., Orazem, J., & Sabol, F. (2022). Effects of resistance training performed to repetition failure or non-failure on muscular strength and hypertrophy: A systematic review and meta-analysis. *Journal of Sport and Health Science*, 11(2), 202–211. 10.1016/j.jshs.2021.01.007 33497853 PMC9068575

[ref10] Halson, S. L. (2014). Monitoring Training Load to Understand Fatigue in Athletes. *Sports Medicine*, 44(S2), 139–147. 10.1007/s40279-014-0253-z PMC4213373 25200666

[ref11] Helms, E. R., Cronin, J., Storey, A., & Zourdos, M. C. (2016). Application of the Repetitions in Reserve-Based Rating of Perceived Exertion Scale for Resistance Training. *Strength and Conditioning Journal*, 38(4), 42–49. 10.1519/SSC.0000000000000218 27531969 PMC4961270

[ref12] Impellizzeri, F. M., Marcora, S. M., & Coutts, A. J. (2019). Internal and External Training Load: 15 Years On. *International Journal* of *Sports Physiology* and *Performance*, 14(2), 270–273. 10.1123/ijspp.2018-0935 30614348

[ref13] Kraemer, W. J., & Ratamess, N. A. (2004). Fundamentals of Resistance Training: Progression and Exercise Prescription. *Medicine & Science in Sports & Exercise*, 36(4), 674–688. 10.1249/01.MSS.0000121945.36635.61 15064596

[ref14] Mansfield, S. K., Peiffer, J. J., Hughes, L. J., & Scott, B. R. (2020). Estimating Repetitions in Reserve for Resistance Exercise: An Analysis of Factors Which Impact on Prediction Accuracy. *Journal of Strength and Conditioning Research*, 10.1519/JSC.0000000000003779. Advance online publication. 10.1519/JSC.0000000000003779 32881842

[ref15] Martinez-Canton, M., Gallego-Selles, A., Gelabert-Rebato, M., Martin-Rincon, M., Pareja-Blanco, F., Rodriguez-Rosell, D., Morales-Alamo, D., Sanchis-Moysi, J., Dorado, C., Gonzalez-Badillo, J. J., & Calbet, J. A. L. (2021). Role of CaMKII and sarcolipin in muscle adaptations to strength training with different levels of fatigue in the set. *Scandinavian Journal of Medicine & Science in Sports*, 31(1), 91–103. 10.1111/sms.13828 32949027

[ref16] Pareja-Blanco, F., Alcazar, J., Sánchez-Valdepeñas, J., Cornejo-Daza, P. J., Piqueras-Sanchiz, F., Mora-Vela, R., Sánchez-Moreno, M., Bachero-Mena, B., Ortega-Becerra, M., & Alegre, L. M. (2020). Velocity Loss as a Critical Variable Determining the Adaptations to Strength Training. *Medicine & Science in Sports & Exercise*, 52(8), 1752–1762. 10.1249/MSS.0000000000002295 32049887

[ref17] Pareja-Blanco, F., Rodríguez-Rosell, D., Sánchez-Medina, L., Gorostiaga, E., & González-Badillo, J. (2014). Effect of Movement Velocity during Resistance Training on Neuromuscular Performance. *International Journal of Sports Medicine*, 35(11), 916–924. 10.1055/s-0033-1363985 24886926

[ref18] Pareja-Blanco, F., Rodríguez-Rosell, D., Sánchez-Medina, L., Sanchis-Moysi, J., Dorado, C., Mora-Custodio, R., Yáñez-García, J. M., Morales-Alamo, D., Pérez-Suárez, I., Calbet, J. A. L., & González-Badillo, J. J. (2017). Effects of velocity loss during resistance training on athletic performance, strength gains and muscle adaptations. *Scandinavian Journal of Medicine & Science in Sports*, 27(7), 724–735. 10.1111/sms.12678 27038416

[ref19] Rhea, M. R., Alvar, B. A., Burkett, L. N., & Ball, S. D. (2003). A Meta-analysis to Determine the Dose Response for Strength Development. *Medicine & Science in Sports & Exercise*, 35(3), 456–464. 10.1249/01.MSS.0000053727.63505.D4 12618576

[ref20] Rodiles-Guerrero, L., Bachero-Mena, B., & Sánchez-Moreno, M. (2026). Impact of Prior Fatigue on Velocity Loss as a Set-Termination Criterion in the Bench Press Exercise. *Journal of Human Kinetics*, 101, 35–48. 10.5114/jhk/214212 42051618 PMC13112160

[ref21] Rodríguez-Rosell, D., Yáñez-García, J. M., Mora-Custodio, R., Torres-Torrelo, J., Ribas-Serna, J., & González-Badillo, J. J. (2020). Role of the Effort Index in Predicting Neuromuscular Fatigue During Resistance Exercises. *Journal of Strength and Conditioning Research*, 10.1519/JSC.0000000000003805. Advance online publication. 10.1519/JSC.0000000000003805 32868675

[ref22] Sánchez-Medina, L., & González-Badillo, J. J. (2011). Velocity Loss as an Indicator of Neuromuscular Fatigue during Resistance Training. *Medicine & Science in Sports & Exercise*, 43(9), 1725–1734. 10.1249/MSS.0b013e318213f880 21311352

[ref23] Sánchez-Moreno, M., Rendeiro-Pinho, G., Mil-Homens, P. V., & Pareja-Blanco, F. (2021). Monitoring Training Volume Through Maximal Number of Repetitions or Velocity-Based Approach. *International Journal of Sports Physiology and Performance*, 16(4), 527–534. 10.1123/ijspp.2020-0214 33406485

[ref24] Schoenfeld, B. J. (2010). The Mechanisms of Muscle Hypertrophy and Their Application to Resistance Training. *Journal of Strength and Conditioning Research*, 24(10), 2857–2872. 10.1519/JSC.0b013e3181e840f3 20847704

[ref25] Schoenfeld, B. J., Grgic, J., Ogborn, D., & Krieger, J. W. (2017). Strength and Hypertrophy Adaptations Between Low-vs. High-Load Resistance Training: A Systematic Review and Meta-analysis. *Journal of Strength and Conditioning Research*, 31(12), 3508–3523. 10.1519/JSC.0000000000002200 28834797

[ref26] Stone, M. H., O’Bryant, H. S., Schilling, B. K., Johnson, R. L., Pierce, K. C., Haff, G. G., & Koch, A. J. (1999). Periodization: Effects Of Manipulating Volume And Intensity. Part 1: *Strength and Conditioning Journal*, 21(2), 56.

[ref27] Stone, M. H., Stone, M., & Sands, W. A. (2007). Principles and practice of resistance training. Human Kinetics.

[ref28] Suchomel, T. J., Nimphius, S., Bellon, C. R., & Stone, M. H. (2018). The Importance of Muscular Strength: Training Considerations. *Sports Medicine*, 48(4), 765–785. 10.1007/s40279-018-0862-z 29372481

[ref29] Wernbom, M., Augustsson, J., & Thomee, R. (2007). The Influence of Frequency, Intensity, Volume and Mode of Strength Training on Whole Muscle Cross-Sectional Area in Humans. *Sports Medicine*, 37(3), 225–264. 10.2165/00007256-200737030-00004 17326698

[ref30] Zatsiorsky, V. M., Kraemer, W. J., & Fry, A. C. (2021). *Science and practice of strength training* (3rd ed. ed.). Human Kinetics.

[ref31] Zourdos, M. C., Klemp, A., Dolan, C., Quiles, J. M., Schau, K. A., Jo, E., Helms, E., Esgro, B., Duncan, S., Garcia Merino, S., & Blanco, R. (2016). Novel Resistance Training–Specific Rating of Perceived Exertion Scale Measuring Repetitions in Reserve. *Journal of Strength and Conditioning Research*, 30(1), 267–275. 10.1519/JSC.0000000000001049 26049792

